# Process design of SSCF for ethanol production from steam-pretreated, acetic-acid-impregnated wheat straw

**DOI:** 10.1186/s13068-016-0635-6

**Published:** 2016-10-18

**Authors:** Pia-Maria Bondesson, Mats Galbe

**Affiliations:** Department of Chemical Engineering, Lund University, P.O. Box 124, 221 00 Lund, Sweden

**Keywords:** Acetic acid, Wheat straw, Steam pretreatment, Ethanol, Simultaneous saccharification and co-fermentation (SSCF), Fed-batch, *Saccharomyces cerevisiae*, Xylose fermentation

## Abstract

**Background:**

Pretreatment is an important step in the production of ethanol from lignocellulosic material. Using acetic acid together with steam pretreatment allows the positive effects of an acid catalyst to be retained, while avoiding the negative environmental effects associated with sulphuric acid. Acetic acid is also formed during the pretreatment and hydrolysis of hemicellulose, and is a known inhibitor that may impair fermentation at high concentrations. The purpose of this study was to improve ethanol production from glucose and xylose in steam-pretreated, acetic-acid-impregnated wheat straw by process design of simultaneous saccharification and co-fermentation (SSCF), using a genetically modified pentose fermenting yeast strain *Saccharomyces cerevisiae*.

**Results:**

Ethanol was produced from glucose and xylose using both the liquid fraction and the whole slurry from pretreated materials. The highest ethanol concentration achieved was 37.5 g/L, corresponding to an overall ethanol yield of 0.32 g/g based on the glucose and xylose available in the pretreated material. To obtain this concentration, a slurry with a water-insoluble solids (WIS) content of 11.7 % was used, using a fed-batch SSCF strategy. A higher overall ethanol yield (0.36 g/g) was obtained at 10 % WIS.

**Conclusions:**

Ethanol production from steam-pretreated, acetic-acid-impregnated wheat straw through SSCF with a pentose fermenting *S. cerevisiae* strain was successfully demonstrated. However, the ethanol concentration was too low and the residence time too long to be suitable for large-scale applications. It is hoped that further process design focusing on the enzymatic conversion of cellulose to glucose will allow the combination of acetic acid pretreatment and co-fermentation of glucose and xylose.

## Background

To meet the challenges of a growing population and increasing energy demand, and the need to reduce greenhouse gas emissions, research has turned towards various kinds of biofuels and biorefinery processes. One such biofuel is bioethanol. A number of commercial biorefinery ethanol plants using lignocellulosic material, such as wheat straw and corn stover, have recently been opened, for example, Beta Renewable’s commercial plant [1] in Crescentino, Italy, Poet-DSM’s Project Liberty commercial cellulosic ethanol plant in Emmetsburg, Iowa, USA [2], and Abengoa’s commercial plant in Hugoton, Kansas, USA [3]. However, there is still a need to improve the process in order to increase cost efficiency. Two areas that can be improved in the ethanol process are the pretreatment step and the subsequent combined hydrolysis and fermentation step.

One of the most common pretreatment methods used to produce ethanol from lignocellulosic material is steam pretreatment, either using steam only or combined with an acid catalyst, e.g. sulphuric acid [4]. Several studies have shown that the use of sulphuric acid as a catalyst during steam pretreatment improves the ethanol yield, decreases the amount of degradation products formed, and reduces the residence time and temperature required [5–7]. A drawback of sulphuric acid is, however, that it is environmentally harmful, and must therefore be removed or recycled in downstream processes. This can be costly, and the use of sulphuric acid during pretreatment is thus not the optimal solution. Acetic acid has been proposed as an alternative to sulphuric acid in several studies [8–10]. The ethanol yields obtained using acetic acid in pretreatment have been shown to be higher than when using steam only [8, 9]. In addition, acetic acid can be converted into biogas by the treatment of waste liquid streams of the process, it is less environmentally harmful than sulphuric acid, and it can be easily handled in downstream processes [8].

One common method of producing ethanol from pretreated biomass is by means of combined enzymatic hydrolysis and fermentation, for example, using simultaneous saccharification and fermentation (SSF). Compared with separate enzymatic hydrolysis and fermentation (SHF), SSF has been reported to result in higher overall ethanol yields [11, 12]. Different process options have been investigated in efforts to obtain high ethanol yields and ethanol concentrations with SSF, including (i) fed-batch SSF with pre-hydrolysis prior to SSF, instead of batch SSF [13–17]; (ii) increasing the content of water-insoluble solids (WIS) [18–20]; and (iii) co-fermentation of pentoses, mainly xylose, together with glucose (SSCF) [13, 16, 21–23]. One of the challenges in SSCF is that a microorganism that can ferment not only glucose, but also xylose, is needed. Wild-type yeast, *Saccharomyces cerevisiae*, cannot do this. During recent decades, extensive research has been carried out on alternative organisms such as bacteria [24, 25], naturally occurring xylose-fermenting yeast strains [26, 27], and genetically modified yeast strains [13, 23]. A great deal of attention has been devoted to genetically modified *S. cerevisiae* strains [28]. However, one problem associated with these organisms is that they are not as tolerant to the process environment as the original *S. cerevisiae* [28].

In order to reduce production costs, it is important that the raw material is exploited to its full extent, in order to optimize the energy output of the overall process. In earlier studies, where acetic acid was used for pretreatment, ethanol was produced from glucose, while the xylose fraction was used for biogas production [8, 29]. In that process, most of the raw material (corn stover and wheat straw) was converted into useful energy. However, using techno-economic calculations, Joelsson et al. showed that the process would be more economically feasible if ethanol could also be produced from xylose [29]. A problem with this approach is that acetic acid, which is also present in the hemicellulose and is released in the pretreatment and hydrolysis steps, has been shown to have a negative impact on fermentation at high concentrations [30, 31]. Since organisms other than wild-type *S. cerevisiae* are either more sensitive to acetic acid or produce less ethanol, it has been difficult to combine acetic acid pretreatment and SSCF. In an earlier study, it was found to be difficult to increase the ethanol concentration, despite the fact that xylose-to-ethanol conversion was obtained in some SSCF strategies [8]. Since then, new yeast strains have been constructed, which it is hoped will facilitate the development of a process in which both acetic acid pretreatment and SSCF can be utilized.

The main purpose of this study was to design a process with the potential to reduce the cost of producing ethanol from lignocellulosic material. The goal was to combine acetic acid steam pretreatment and ethanol production from both glucose and xylose. To the best of our knowledge, this has not been previously achieved. The genetically modified *S. cerevisiae* strain, KE6-12b, was used as a possible yeast for co-fermentation of xylose and glucose. In the first step, the potential of KE6-12b to ferment xylose in the pretreatment liquid, and in SSCF where both the liquid and the solid fractions of the pretreatment step were present, was evaluated. This was done to determine the ability of this yeast strain to produce ethanol in an acetic-acid-rich environment and to investigate the ethanol yield and concentration that could be obtained using different pretreatment fractions. In the second step, attention was focused on achieving high ethanol yields in combination with short residence times during SSCF, in order to decrease the ethanol production cost. This was investigated by comparing the performance of different SSCF configurations. Furthermore, the WIS content and the yeast cell concentration during SSCF were increased in an attempt to increase the ethanol concentration.

## Results and discussion

### Investigation of liquid fermentation with and without pre-hydrolysis

**Fig. 1:**
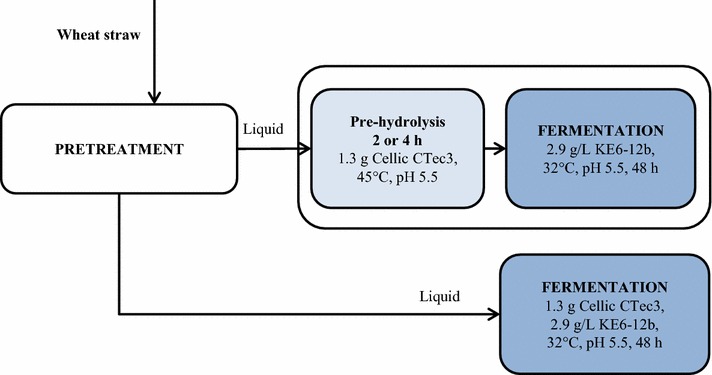
Schematic description of the liquid fermentation process for experiments with and without pre-hydrolysis

Figure 1 illustrates the liquid fermentation process investigated. In the first step, the ability of the yeast to ferment sugars, mainly xylose, to ethanol in the pretreatment liquid was investigated. The liquid was diluted to correspond to the same dilution, having the same total reactor loading as in the liquid fermentation step of the SSCF configurations discussed below. A small amount (1.3 g in 888 g liquid, which corresponds to 20 % of the total addition in the SSCF configurations) of enzymes was added, since a large proportion of the sugars in the liquid were oligomers (85 and 73 % of detected glucose and xylose, respectively). Since one of the goals of the study was to achieve a short residence time, the effect of a pre-hydrolysis step was investigated. This step lasted for either 2 or 4 h at a higher temperature (45 °C), before the temperature was reduced to 32 °C and the yeast was added.

**Fig. 2:**
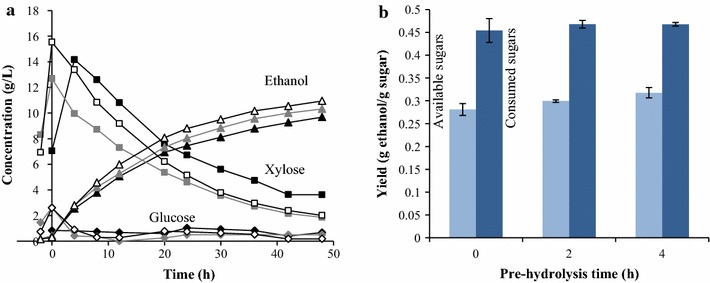
**a** Concentrations of ethanol, xylose, and glucose during liquid fermentation with no pre-hydrolysis (*black*) and with pre-hydrolysis for 2 (*grey*) and 4 (*white*) h. **b** Overall and metabolic ethanol yield (g/g) based on available sugars (*pale blue*) and consumed sugars (*dark blue*), respectively, after liquid fermentation with no pre-hydrolysis (0 h) and with pre-hydrolysis for 2 and 4 h. The *error bars* represent the highest and lowest results of duplicate experiments

Figure 2 shows the average results of the liquid fermentation experiments, where it can be seen that this strain of *S. cerevisiae* can utilize xylose, despite the presence of acetic acid (Fig. 2a). The initial concentration of acetic acid was 3.5 g/L, and rose to 5–6 g/L after 4 h of fermentation, finally reaching a concentration of 6.3 g/L. A small increase in ethanol yield was seen with increasing pre-hydrolysis time, based on the theoretical amount of monomer and oligomer sugars available (overall yield) (Fig. 2b). No such trend was found when the yield was based on the amount of fermentable sugars consumed (metabolic yield). Most of the consumed sugars were converted to ethanol (around 90 % of theoretical yield) in all cases. The similar metabolic yields indicate that the fermentation yield is the same regardless of the amount of available sugars, and that the enzyme activity governs the overall ethanol yield. The overall yield was higher when pre-hydrolysis was included, as the enzyme activity increases with temperature. The xylose consumption is slightly higher and faster with pre-hydrolysis than without. Pre-hydrolysis thus leads to a higher overall yield in a shorter time in liquid fermentation.

### Investigation of different SSCF configurations

**Fig. 3:**
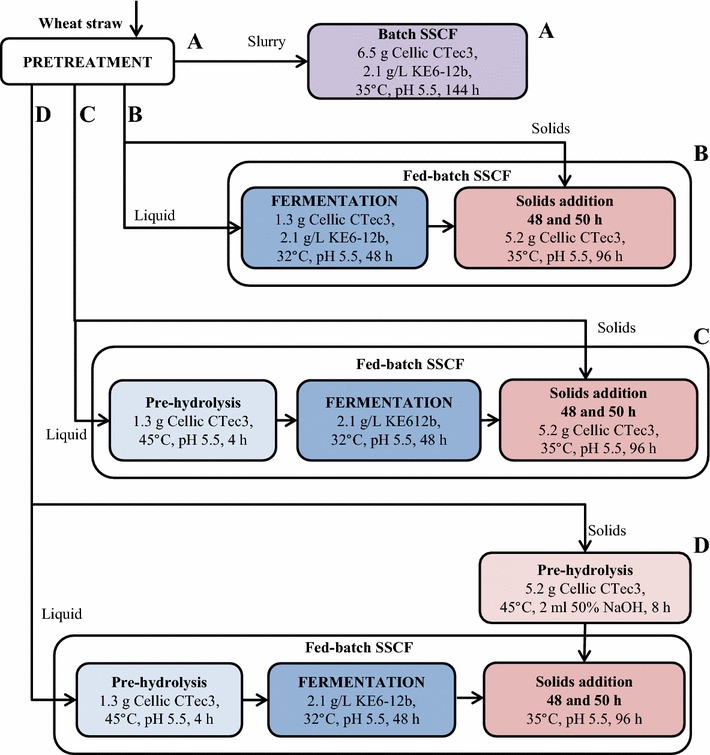
Overview of the four SSCF configurations investigated. **A** Batch SSCF (base case). **B** Fed-batch SSCF of the liquid, followed by the addition of solids at 48 and 50 h. **C** As in **B**, but with 4-h pre-hydrolysis of the liquid fraction. **D** As in **C**, but with 8-h pre-hydrolysis of the solid fraction

Different SSCF configurations were used to investigate the ability of the yeast to ferment sugars in environments containing both the liquid and the solids from the pretreatment step. Figure 3 illustrates the four configurations investigated. The total reactor loading was in all SSCF configurations 1.2 kg. Batch SSCF (Configuration A) was performed as the base case to take into account external effects, such as the inhibitors already present in the liquid and solid fractions. The batch SSCF experiment was also performed to allow comparison of the performance of KE6-12b with other *S. cerevisiae* strains used in previous studies. Three fed-batch SSCF configurations were investigated in an attempt to obtain high ethanol yields. Configuration B involved the fermentation of the liquid without any pre-hydrolysis, followed by the addition of half of the solids together with the enzymes after 48 and half of the solids after 50 h. Even though the time between the additions of solids was only 2 h, a clear viscosity reduction was seen before the second addition was made. Configuration C was the same as B, but the liquid was subjected to 4 h of pre-hydrolysis. The final configuration investigated (D) was the same as Configuration C, but included 8 h of pre-hydrolysis of the solids before they were added to the liquid fraction.

**Fig. 4:**
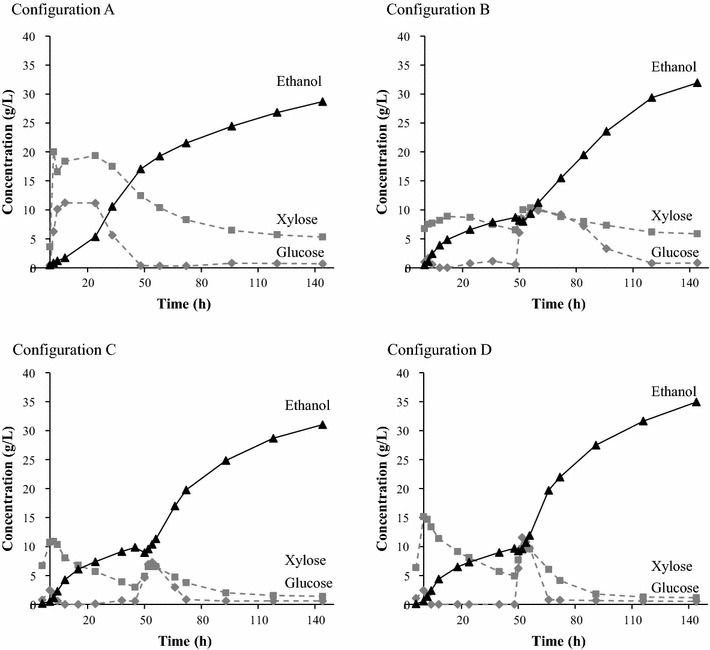
Ethanol and sugar concentrations during SSCF using the Configurations *A*–*D* as defined in Fig. 3

Figure 4 shows the average results of SSCF using the different configurations. Batch SSCF (Configuration A) resulted in ethanol production from both glucose and xylose (Fig. 4). It is difficult to make comparisons between different studies since the pretreatment method and the yeast strain used vary, and will affect the results. Conflicting results have been reported from previous studies concerning the production of ethanol from both glucose and xylose in batch SSCF [8, 13, 21, 22, 32]. In one study reported by Bondesson et al. using *S. cerevisiae* KE6-12 [8], no xylose was consumed during batch SSCF, while in another study performed by Koppram et al. also using *S. cerevisiae* KE6-12 [21] ethanol was produced from xylose but more xylose was found after SSCF and the overall ethanol yield was lower than in the present study (0.28 compared with 0.30 g/g). Moreno et al. reported that it was necessary to detoxify the slurry to obtain any ethanol production, also using *S. cerevisiae* KE6-12 [22]. Zhu et al. used *S. cerevisiae* SyBE005 and found it necessary to use high yeast concentrations and pre-hydrolysis to achieve successful xylose utilization and high ethanol concentration in batch SSCF [32]. Olofsson et al. used *S. cerevisiae* TMB3400 and low WIS concentration (7 %) to obtain ethanol yields of 0.38 g/g in batch SSCF [13]. The acetic acid concentration in the present study was only 3.5 g/L at the start of SSCF, despite the fact that acetic acid was added during pretreatment. This concentration is lower than in the previous studies mentioned above, except in that by Moreno et al. [22]. However, the acetic acid pretreatment used in the present study is mild, and all acetate present in the hemicellulose is not released after pretreatment. The concentration of acetic acid had increased to 7 g/L by the end of SSCF. In the present study, it was evident that KE6-12b utilized both glucose and xylose in batch mode at 10 % WIS, at a moderate yeast concentration of 2.1 g/L, without any need for detoxification, despite the fact that acetic acid was added as a catalyst in the pretreatment step. This shows that KE6-12b performs reasonably well, also in batch SSCF.

**Fig. 5:**
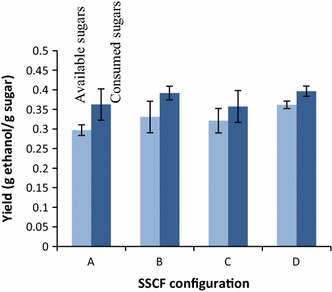
Overall and metabolic ethanol yield (g/g) based on available sugars and consumed sugars, respectively, for the four SSCF configurations defined in Fig. 3. The *error bars* represent the highest and lowest results of duplicate experiments

Ascan be seen from Fig. 5, there is a difference in the overall ethanol yield between batch and fed-batch SSCF. Although the error bars in Configurations B and C are slightly overlapping Configuration A, the results indicate that the potential for reaching higher ethanol yield is higher with fed-batch than batch SSF. There is no clear difference between the fed-batch configurations (B, C, and D), and an overall ethanol yield above 0.35 g/g (based on available sugars) was observed in at least one of the duplicate experiments. This indicates that the way in which the fed-batch SSCF is performed is less important than the fed-batch strategy itself. However, as shown in Fig. 4, the xylose consumption rates differ between the various configurations. The consumption rate of xylose before the addition of solid materials was higher using Configurations C and D. The rapid consumption of glucose facilitates uptake and utilization of xylose by converting most of the xylose in the liquid. Because of the higher xylose consumption rate prior to addition of solids material, more xylose is consumed overall in Configurations C and D. The difference between batch and fed-batch SSCF are in line with those reported in earlier studies [13, 21, 22], but the difference in overall ethanol yield between batch and fed-batch SSCF is smaller in the present study. One explanation of this smaller difference could be the good performance of the yeast strain. There are several possible explanations of the positive effects of fed-batch compared with batch SSCF. One may be that since the material is added in portions (one liquid and two solids additions), the amount of inhibitors in relation to the amount of yeast is initially low. A gradual increase in the amount of inhibitors compared to the amount of yeast might lead to a lower toxic effect due to the adaptation of the yeast to the environment, which is not possible to the same extent using batch fermentation [22, 23]. Another explanation may be the gradual decrease in viscosity due to enzymatic hydrolysis before more solids are added [33]. The yeast seems to adapt quickly to the environment, in which case it is more likely that the small difference between the overall ethanol yields in the batch and fed-batch configurations in this study is due to more efficient mass transfer, rather than inhibition of yeast.

In all cases, using batch or fed-batch configurations, at least one of the duplicate experiments reached a metabolic ethanol yield of 0.40 g/g consumed sugar. These findings support the hypothesis that the difference in overall yield between batch and fed-batch is to some extent explained by viscosity and mass transfer, in terms of less efficient stirring and enzymatic hydrolysis, rather than inhibition of the yeast. The metabolic ethanol yield obtained using only the liquid from pretreatment was 0.47 g/g, and thus the addition of solids reduced the metabolic ethanol yield. The amount of glycerol formed, based on consumed sugars, did not differ between SSCF and liquid fermentation (0.04–0.10 and 0.06–0.13 g/g, respectively), and almost no xylitol was formed in either case (maximum 1.0 and 0.5 g/L, respectively), so formation of these compounds cannot explain the different metabolic ethanol yields. Increased inhibition due to the addition of solids has also been observed in previous studies [34–36]. However, the compounds responsible for the increase in inhibition were not identified in the studies.

### Increasing the WIS content

The final ethanol concentration using the process configurations discussed above did not exceed 36 g/L, and the average concentration was about 33 g/L. A concentration of at least 5 % ethanol based on volume [37], which corresponds to a concentration of 39 g/L, is desirable to limit the energy required in the distillation step. Therefore, Configuration C was investigated at a higher WIS concentration (without removing any liquid from the pretreated material). The WIS content after pretreatment was 11.9 %. The highest possible WIS content was found to be 11.7 % after addition of yeast, enzymes, and nutrients.

**Fig. 6:**
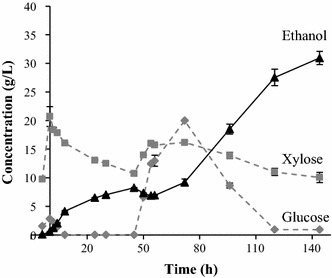
Concentration of ethanol, xylose, and glucose during fed-batch SSCF (Configuration C) with pre-hydrolysis of the liquid fraction at a total WIS of 11.7 %. The *error bars* represent the highest and lowest results of duplicate experiments

As can be seen from Fig. 6, KE6-12b has the ability to ferment both glucose and xylose despite the higher inhibitor concentration. However, the fermentation rate was much lower than at the lower WIS content. This resulted in a lower final ethanol concentration (maximum 32 g/L) and overall yield (0.28 g/g based on available sugars) than in the fed-batch experiments using 10 % WIS. There are some possible explanations to this decrease in concentration and yield. Either the yeast or the enzymes are affected by the increase in WIS content or a combination of both. The lower fermentation rate indicates that the yeast is affected. The decrease in fermentation rate and yield may be that the amount of yeast added was too low. Increasing the WIS content increased the amount of inhibitors, which may have inhibited the yeast, resulting in lower ethanol production.

### Increasing the yeast concentration

Two sets of experiments were conducted to investigate whether the amount of yeast was a limiting factor during fed-batch SSCF. Configuration C was performed with 10 and 11.7 % WIS with increased yeast addition (4.3 g/L) compared to the previous experiments (2.1 g/L).

**Fig. 7:**
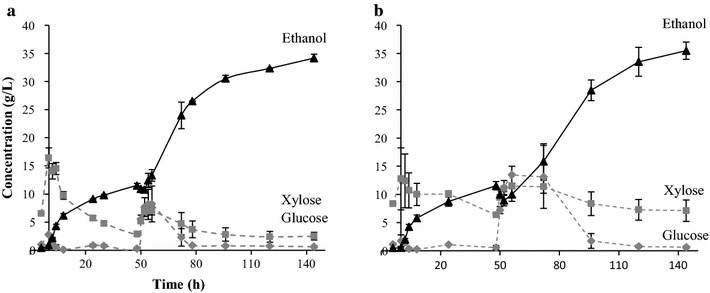
Concentration of ethanol, xylose, and glucose during fed-batch SSCF with pre-hydrolysis of the liquid fraction and 4.3 g yeast/L at a total WIS content of **a** 10 % and **b** 11.7 %. The *error bars* represent the highest and lowest results of duplicate (10 % WIS) and triplicate (11.7 % WIS) experiments

Figure 7 shows the results from SSCF using increased yeast addition. The results showed that the variation in ethanol concentration and yield at 10 % WIS was smaller using the higher yeast concentration (34.2 ± 0.7 g/L and 0.35 ± 0.007 g/g) compared with the lower yeast concentration (0.32 ± 3.0 g/L and 0.32 ± 0.03 g/g). At the higher WIS concentration (Fig. 7b), there was an improvement in ethanol production with the higher yeast concentration, showing an ethanol concentration of 35.5 ± 2.0 g/L (overall yield of 0.31 ± 0.01 g/g) instead of 32 ± 1.2 g/L (overall yield of 0.28 ± 0.01 g/g). In addition, the fermentation rate was higher (cf. Figs. 6, 7b). Thus, the ethanol concentration increased at 11.7 % WIS by the greater yeast addition, and the smaller yeast addition in the 10 % WIS experiments was one of the reasons to the lower yield and fermentation rate. In the experiment with the higher WIS content, the xylose concentration was still above 5 g/L (Fig. 7b). This indicates that the effect of inhibitors on xylose fermentation is higher at 11.7 % WIS than at 10 % WIS also when increasing the yeast concentration.

There is potential to obtain higher ethanol concentrations and yield, as the highest overall yield obtained was only 71 % (0.36 g/g) of the theoretical (Fig. 5). The yeast was able to ferment xylose and glucose despite the use of acetic acid as a catalyst during pretreatment. However, the effect of inhibitors in the solid and the liquid fractions was evident with increasing WIS. Since the overall yield is only moderate, it can be assumed that it is possible to obtain higher ethanol concentrations and therefore higher overall yields. This is probably due to low enzymatic hydrolysis yield. This is also confirmed by the metabolic yield. Since this yield is approximately 0.04–0.07 g/g higher than the overall yield in all experiments, it can be assumed that a higher overall yield would have been obtained if better enzymatic hydrolysis could be achieved, i.e. if more monomer sugars had been available. The results of the SSCF experiments show that both xylose and glucose are consumed at 10 % WIS (Fig. 4 Configurations C and D, Fig. 7a), confirming that enzymatic hydrolysis limits the ethanol production. This could be overcome by extending the residence time. This was confirmed in two experiments in which the residence time was extended. The end concentration of ethanol increased from 34.9 to 37.2 g/L at 10 % WIS with another 24 h of residence time. The residence time was extended with 96 h at 11.7 % WIS and showed that it was possible to obtain an end concentration of ethanol of 40 g/L. However, such a long residence time is not economically feasible [29].

In summary, the results of this study show that the rate (but also the yield) of enzymatic hydrolysis must be improved to obtain higher ethanol concentrations and yields. One reason for the low conversion rate could be the low severity of the pretreatment, resulting in less cellulose being available for enzymatic hydrolysis. One way of overcoming this could be to use two-step pretreatment, in which the solids are treated again after separation from the liquid [38]. This would make more cellulose accessible, without degrading too much xylose. This would be feasible as the pretreatment slurry is already separated into a solid and a liquid fraction. Other strategies to improve hydrolysis, as alternatives to two-step pretreatment, include increasing the enzyme concentration, and detoxifying the slurry by adding laccase enzymes [22]. However, detoxification of the slurry will only be beneficial if lignin is responsible for the lower yield. The disadvantage of these alternatives is the higher operational cost of the process. Another alternative may be to employ an enzyme feeding strategy. This has been shown to have a positive effect on SSF and SSCF in previous studies [16, 18, 39].

## Conclusions

The overall goal of this work was to show that acetic acid steam pretreatment and ethanol production using SSCF was possible. We have demonstrated this using the genetically modified yeast strain *S. cerevisiae* KE6-12. Co-fermentation of glucose and xylose was observed during the fermentation of pretreatment liquid and during batch and fed-batch SSCF of the whole pretreated material. The highest ethanol concentration obtained was 37.5 g/L, corresponding to an overall ethanol yield of 0.32 g/g. This was achieved using fed-batch SSCF with 11.7 % WIS and a relatively high yeast concentration (4.3 g/L). The highest overall ethanol yield, 0.36 g/g, was obtained at 10 % WIS and 2.1 g/L yeast. Although the results were reasonably good, the ethanol concentration is still slightly low for energy-efficient distillation. In addition, the residence time for SSCF is still too long. Therefore, improvements are required in the process involving the combination of acetic acid steam pretreatment and SSCF to achieve a commercially viable process for the production of ethanol. Enzymatic hydrolysis and the pretreatment step were identified as limiting factors, and in order to improve the process design future research should be directed towards strategies to increase the rate and yield of enzymatic hydrolysis.

## Methods

### Raw material and pretreatment

Wheat straw, locally harvested in August 2013 and dried on field (Johan Håkansson *Lantbruksprodukter*, Lunnarp, Sweden), was chopped into pieces up to 50 mm long using a knife mill (Retsch GmbH, Haan, Germany). The dry matter (DM) content of the wheat straw was measured by drying the material in an oven at 105 °C until constant weight was obtained, and was found to be 90 %. The composition was determined using standardized analytical procedures from the National Renewable Energy Laboratory (NREL) [40], and is given in Table 1.

**Table 1:** Composition of wheat straw expressed as percentage of dry matter (average and standard deviation of three measurements)

	Ave. content (%)	SD
Glucan	29.3	1.2
Xylan	21.6	1.9
Galactan	0.3	0.0
Arabinan	3.2	0.2
Mannan	0.1	0.1
Lignin^a^	27.5	1.3

^a^Acid-soluble and -insoluble lignin and lignin ash are included

The wheat straw was soaked for 1 h in warm tap water with 1 % (by weight) acetic acid solution at room temperature in sealed buckets. The ratio between the wheat straw and the liquid was 1:20 by weight. After 1 h, the soaked material was dewatered to a DM content of 45–55 % (by weight) in a filter press (Tinkturenpressen HP5 M, Fischer Maschinen-fabrik GmbH, Burgkunstadt, Germany), and then stored at room temperature in sealed buckets overnight until pretreated. The impregnated material was steam pretreated in a steam pretreatment unit using a 10-L batch reactor described previously [8], at 190 °C for 10 min. The steam-pretreated slurry was thoroughly mixed and stored at 4 °C. The structural carbohydrates, lignin, and ash content in the water-insoluble solids and the sugars, by-products (acetic acid), and degradation products [furfural and HMF (5-hydroxymethyl-2-furaldehyde)] in the liquid fraction were determined using standardized NREL analytical procedures [40, 41]. The total DM content was measured by drying the material in an oven at 105 °C until constant weight was obtained. The WIS content of the pretreated material was determined using the method developed by Weiss et al. [42]. The composition of the pretreated wheat straw is given in Table 2.

**Table 2:** Composition of steam-pretreated wheat straw (average and SD of three measurements)

	Ave. content	SD
DM (%)	16.7	0.3
WIS (%)	11.9	0.4
Content in solid fraction (% WIS)		
Glucan	44.7	1.5
Xylan	11.9	0.8
Lignin^a^	32.0	0.9
Content in liquid fraction (g/L)		
Glucose^b^	6.5 (1.0)	0.0
Xylose^b^	36.3 (9.9)	0.1
Acetic acid	4.6	0.0
Furfural	1.5	0.0
HMF	0.2	0.0

^a^Acid-soluble and -insoluble lignin and lignin ash are included

^b^Both monomeric and oligomeric forms are included; concentration of monomeric sugars are in parenthesis

Most of the pretreatment slurry was separated into a liquid and a solid fraction using a filter press. The rest of the slurry was used for batch SSCF experiments. The liquid fraction was then filtered using a vacuum filtration unit to remove the particles from the liquid. The particles were mixed with the solid fraction. The WIS content of the solid fraction was 40 %, and both the liquid and solid fractions were stored at 4 °C.

### Yeast strain and cultivation

The yeast strain used was the pentose fermenting strain *S. cerevisiae* KE6-12b [43] provided by Taurus Energy AB (Lund, Sweden).

Cultivation was initiated by adding 50 μL of the yeast strain from a −80 °C glycerol stock yeast culture to a 50-mL Falcon™ tube containing 10 mL sterile medium. The composition of the medium was 30 g/L glucose, 5 g/L Formedium™ CAS01, 5 g/L (NH_4_)_2_SO_4_, 3 g/L H_2_KPO_4_, and 0.5 g/L MgSO_4_·7H_2_O. The medium also contained 2 mL/L trace metal solution and 1 mL/L vitamin solution prepared as described by Taherzadeh et al. [44]. The tube with the culture was incubated at 30 °C for 24 h on a rotary shaker. Three tubes containing cultivation medium were prepared for each reactor.

The culture was transferred to a 500-mL glass Erlenmeyer flask after 24 h, containing a total volume of 150 mL after addition of the pre-culture. The composition of the sterile medium was 30 g/L glucose, 60 g/L xylose, 30 % (by weight) sterile filtered pretreatment liquid, 10 g/L (NH_4_)_2_SO_4_, 6 g/L H_2_KPO_4_, 1 g/L MgSO_4_·7H_2_O, 2 mL/L trace metal solution, and 1 mL/L vitamin solution. The flask was sealed with a cotton plug and aluminium foil, and incubated at 30 °C for 72 h on a rotary shaker.

After cultivation the cells were harvested using a Jouan C4-12 centrifuge (St Herblain, France) at 4000 rpm for 15 min. The pellet was re-suspended in a mixture of pretreatment liquid and deionized water corresponding to the same mixture ratio as that used during fermentation and SSCF experiments. After re-suspension, the cells were centrifuged again. The time from the end of cultivation to the start of fermentation and SSCF with the harvested cells was less than 2 h. The amount of yeast produced was roughly determined by measuring the absorbance at 600 nm in the different Erlenmeyer flasks and transformed into dry yeast mass using a calibration curve determined by comparing the absorbance and the DM of the yeast cells. This gave an approximate value of the DM which could be used for yeast addition to the fermentation experiments. The actual DM of the yeast was measured by washing a known amount of yeast several times in deionized water and then drying it in an oven at 105 °C until constant weight was obtained. The variation of the yeast concentration was 0.08 g/L for the experiments using the lower yeast concentration (2.1 g/L), and 0.18 g/L in the experiments using the higher yeast concentration (4.3 g/L).

### Enzymes

The enzyme cocktail Cellic CTec3 from Novozymes A/S, Bagsværd, Denmark was used in all fermentation and SSCF experiments.

### Liquid fermentation

The liquid fermentation experiments were performed in duplicate in 2-L fermenters (Infors AG, Bottmingen, Switzerland) with a total loading of 888 g, which was the same as the amount used in the first step of SSCF. A total of 878 g of liquid mixture was used, where 80 % was pretreatment liquid and the rest deionized water. Most of the liquid was poured directly into the fermenter, while some of the liquid was used to dilute the yeast and enzymes before addition. The remaining 10 g consisted of nutrients, enzymes, and yeast. Nutrients ((NH_4_)_2_HPO_4_) were added to a final concentration of 0.68 g/L.

Three kinds of liquid fermentation experiments were performed: fermentation and fermentation with 2- or 4-h pre-hydrolysis (Fig. 1). The nutrients and water were sterilized together with the fermenter, and the pretreatment liquid was added after cooling to room temperature. The pretreatment liquid was not sterilized to avoid degradation of the material and to obtain similar process conditions to those in large-scale applications. A temperature of 45 °C was used for pre-hydrolysis, and 32 °C during fermentation. The stirring rate was set to 300 rpm and the pH was initially adjusted manually to 5.5 by adding 50 % (by weight) NaOH solution. The liquid mixture not added to the reactor was also adjusted to pH 5.5. During liquid fermentation, pH adjustment was carried out automatically with 10 % NaOH. After setting the pH and temperature, 1.3 g of enzymes diluted in some of the liquid mixture was added to the reactor to start pre-hydrolysis for 2 or 4 h. After the pre-hydrolysis phase, the temperature was reduced to 32 °C and yeast corresponding to a concentration of 2.9 g/L was added. Fermentation without pre-hydrolysis was initiated by adding 1.3 g enzymes and the same concentration of yeast as above. The duration of the fermentation phase was 48 h, and samples were taken for HPLC analysis every 4 h during the first 24 h, and every 6 h during the last 24 h. Samples were also taken after enzyme and yeast addition, and at the end of fermentation. After the fermentation period, the fermentation broth was filtered and analysed to obtain the remaining total sugar concentration (both monomers and oligomers) [41], and the yeast concentration was determined by washing and drying the filter cake.

### Batch SSCF

Batch SSCF was performed in duplicate using the same kind of fermenters as those used for liquid fermentation. The total loading was 1.2 kg and the final WIS content 10 %, obtained by dilution with deionized water. Nutrients were added to a final concentration of 0.5 g (NH_4_)_2_HPO_4_/L. The nutrients and the water were sterilized together with the fermenter, and the pretreatment slurry was added after cooling to room temperature. The pretreatment slurry was not sterilized for the same reasons as above. The temperature was set to 35 °C, the stirring rate to 500 rpm, and the pH was initially adjusted manually to 5.5 by adding 50 % (by weight) NaOH solution. During SSCF, pH adjustment was carried out automatically with 10 % NaOH. The experiment was initiated by adding 6.5 g (corresponding to 0.054 g/g WIS) of enzymes and yeast to a concentration of 2.1 g/L, and was run for 144 h (Fig. 3, Configuration A). Samples were taken for HPLC analysis throughout the experiments. Liquid was filtered from the slurry and analysed to obtain sugar concentration remaining after SSCF (both monomers and oligomers).

### Fed-batch SSCF

All fed-batch SSCF experiments were performed in duplicate, (except for the experiment using high WIS and high yeast addition, which was performed in triplicate) using the same kind of fermenters as those used for liquid fermentation. The fed-batch procedure used was intermittent feeding, but referred to as fed-batch SSCF throughout the paper. The total loading was 1.2 kg and the total WIS content 10 %, resulting in a total concentration of yeast and nutrients of 2.1 and 0.5 g/L, respectively, and a total enzyme addition of 6.5 g (corresponding to 0.054 g/g WIS). The first part of fed-batch SSCF followed the same procedure as liquid fermentation with no pre-hydrolysis or 4 h of pre-hydrolysis. After 48 h of fermentation, the temperature was increased to 35 °C and the stirring rate to 500 rpm. Solids were then added in two equal portions 48 and 50 h after yeast addition. The rest of the enzymes (5.2 g) was added together with the first solids addition. The pH was initially manually adjusted to 5.5 with 50 % (by weight) NaOH solution after the solids additions. During SSCF, pH adjustment was carried out automatically with 10 % NaOH. The SSCF was run for 96 h after the first solids addition (Fig. 3, Configurations B and C). Liquid was filtered from the slurry for analysis to obtain the sugar concentration remaining after fed-batch SSCF (monomers and oligomers). Samples for HPLC analysis were taken throughout the experiments.

In one fed-batch SSCF configuration, the solids were pre-hydrolysed together with 5.2 g enzymes before being added to the SSCF fermenter (Fig. 3, Configuration D). Pre-hydrolysis was performed in the same kind of fermenter as those described above, which had been sterilized. The temperature was set to 45 °C and the residence time was 8 h. After 8 h, the material (which was slightly less viscous but still solid) was added to the liquid in two equal portions as described above.

The fed-batch SSCF experiment using 4-h pre-hydrolysis of the liquid fraction (Fig. 3, Configuration C) was performed with a higher WIS. The total WIS content was 11.7 %; no water was added, and 17 % extra pretreatment liquid and solids were added in each step. The total loading was still 1.2 kg, and the total enzyme addition was 7.6 g (corresponding to 0.054 g/g WIS) (1.5 g during the pre-hydrolysis of the liquid and 6.1 g during solids addition after 48 h).

In two experiments, based on Configuration C in Fig. 3, the yeast addition was increased to a final concentration of 4.3 g/L. Otherwise, the conditions were the same as for the fed-batch SSCF with 4-h pre-hydrolysis with 10 or 11.7 % WIS.

### HPLC analysis

HPLC was used for the analysis of sugars, ethanol, degradation products, and by-products using a chromatographic system equipped with a differential refractive index detector (RID-10A) (both from Shimadzu, Kyoto, Japan). All samples were passed through a filter with a pore diameter of 0.20 µm prior to analysis to remove particles. The filtered samples were stored at −20 °C before analysis. The samples were diluted if necessary, and analysed using an Aminex HPX-87H column (Bio-Rad, Hercules, CA, USA) at 50 °C using 5 mM H_2_SO_4_ as eluent, at a flow rate of 0.5 mL/min, to separate arabinose, ethanol, lactic acid, acetic acid, glycerol, HMF, and furfural. The samples were also analysed using an Aminex HPX-87P column (Bio-Rad) at 85 °C using deionized water as eluent, at a flow rate of 0.5 mL/min, to separate monomeric sugars (glucose, xylose, and galactose) and xylitol. All acidic samples (below pH 5) analysed on this column had been previously neutralized using solid CaCO_3_.

### Yield calculations

Two different ethanol yields were calculated: overall yield and metabolic yield. Overall yield was based on the glucose and xylose available in the WIS as glucan and xylan and the glucose and xylose in both monomeric and oligomeric form available in liquid. Metabolic yield was based on the consumed glucose and xylose. This was calculated by subtraction of the measured amounts of monomeric and oligomeric sugars in the liquid after SSCF from the sugars available in WIS and liquid.

## Abbreviations

DM: dry matter
HMF: 5-hydroxymethyl-2-furaldehyde
NREL: National Renewable Energy Laboratory
SSCF: simultaneous saccharification and co-fermentation
SSF: simultaneous saccharification and fermentation
WIS: water-insoluble solids
